# Non-invasive assessment of ovarian activity in free-ranging eastern black rhinoceros (*Diceros bicornis michaeli*) in Kenya

**DOI:** 10.1093/conphys/coad010

**Published:** 2023-04-17

**Authors:** Maureen W Kamau, Janine L Brown, Nicole Boisseau, Jamie Gaymer, James Hassell, Dino J Martins, Suzan Murray

**Affiliations:** Global Health Program, Smithsonian National Zoo Conservation Biology Institute, Washington DC 20008, USA; Mpala Research Centre, P.O Box 555-10400, Nanyuki, Kenya; Center for Species Survival, Smithsonian National Zoo Conservation Biology Institute, Front Royal, VA 22630, USA; Center for Species Survival, Smithsonian National Zoo Conservation Biology Institute, Front Royal, VA 22630, USA; Ol Jogi Wildlife Conservancy, P.O Box 259-10400, Nanyuki, Kenya; Global Health Program, Smithsonian National Zoo Conservation Biology Institute, Washington DC 20008, USA; Mpala Research Centre, P.O Box 555-10400, Nanyuki, Kenya; Global Health Program, Smithsonian National Zoo Conservation Biology Institute, Washington DC 20008, USA

## Abstract

Eastern black rhinos (*Diceros bicornis michaeli*) are a critically endangered species living in diverse habitats across Africa. In Kenya, once threatened with extinction due to massive poaching pressures, increased protection has resulted in losses being less than 1% annually today. Still, some populations have failed to achieve desired population growth targets. At Ol Jogi Wildlife Conservancy, some individuals are experiencing sub-optimal reproduction based on historical calving records and long inter-calving intervals (>3 years). Hormones drive the reproductive process, so non-invasive assessments of endocrine patterns can be useful indicators of individual reproductive health. In this study, we analysed longitudinal fecal progestagen metabolite (fPM) concentrations in all breeding female eastern black rhinos at Ol Jogi (n = 17) and compared the prevalence of irregular estrous cycles (longer or shorter than 20–40 days) and anestrous periods (interluteal period more than twice the length of a normal follicular phase, i.e. > 10 days) between optimal (inter-calving interval < 3 years) and sub-optimal (>3 years) reproducing individuals. Ten rhinos were pregnant during at least part of the study period. A total of 12 complete cycles were observed in seven females with an average length of 36 ± 3 days and equal numbers of regular and irregular cycles. Single anestrous periods averaging 67 ± 13 days were observed in five females. Surprisingly, a majority of cycles in optimal reproducing individuals were categorized as irregular based on fPM profiles. Overall, results suggest that irregular ovarian activity and isolated bouts of anestrus do not have negative impacts on reproductive performance in this subpopulation at Ol Jogi. A high priority is to continue using noninvasive hormone monitoring to evaluate how ecological or other variables influence reproductive success in this and other eastern black rhino subpopulations in Kenya.

## Teaser

Black rhinos experience suboptimal population growth in Kenya, thus fecal progestagens were measured in 17 females at Ol Jogi Wildlife Conservancy to assess ovarian function. Regular and irregular cycles were observed, however neither were associated with poor reproductive performance. These methods and findings are foundational for future studies.

## Introduction

Poaching for horn constitutes a major threat to the conservation of eastern black rhinos (*Diceros bicornis michaeli*) in the wild. Thus, every birth is important. Further, maintenance of genetic diversity and long-term species survival hinges on maintaining sustainable annual growth rates for semi-captive, sanctuary and wild populations. To that end, monitoring reproductive performance and population demographics is a key element of rhino management both *in situ* and *ex situ* (Milliken *et al.,* 2009; Edwards *et al.,* 2015; Amin *et al.,* 2017). During the early 1970s to late 1980s, major reductions in rhino numbers, from 15 000 to 500 individuals, occurred as a result of poaching in Kenya (Okita-Ouma *et al.,* 2007; Karanja, 2012; Khayale *et al.,* 2020). With increased monitoring and security measures, poaching losses in Kenya now stand at less than 1% annually (Amin *et al.,* 2017), and some populations are increasing at rates of 2.4% to 6% per annum. With > 800 individuals, Kenya now holds 84% of the world’s *in situ* eastern black rhino population (Khayale *et al.,* 2020). However, despite that growth, some populations are well below the desired 5% per annum growth rate target, with suboptimal reproduction being one of the problems affecting sustainability (Khayale *et al.,* 2020). At Ol Jogi Wildlife Conservancy, while the average inter-calving interval of 2.9 years indicates good to moderate fecundity (du Toit, 2006), some individuals are performing sub-optimally, with inter-calving intervals longer than 3 years (Ol Jogi Wildlife Conservancy records). For small populations, every reproductive-age female should reproduce regularly, which does not appear to be the case for all subpopulations in Kenya.

Understanding the reproductive biology and endocrinology of slow-breeding endangered species is a critical component for their effective conservation and a valuable conservation physiology tool for monitoring individual reproductive health (Brown *et al.,* 2001; Herrick, 2019). An endocrine laboratory dedicated to wildlife was recently established at the Mpala Research Centre in Kenya and has demonstrated the feasibility of longitudinally monitoring ovarian cyclicity and diagnosing pregnancy in Kenya's black rhinos (Kamau *et al.,* 2021). Previous studies of eastern black rhinos have identified several factors that can impact reproductive output (Edwards, 2013; Edwards *et al.,* 2015). In particular, irregular ovarian cycles defined as those longer or shorter than 20 to 40 days are prevalent among both captive (Edwards *et al.,* 2015) and wild (Garnier *et al.,* 2002) females, and are associated with reduced fertility. Further, a high incidence of prolonged anestrous periods in female black rhinos has been identified as problematic for captive breeding (Ververs *et al.,* 2015). How or if irregular cycles and anestrus are impacting fertility of Kenya’s wild eastern black rhino population has yet to be determined. Thus, in this study, progestagen metabolites were measured in fecal samples collected longitudinally for 5 to 12 months in all breeding-age female eastern black rhinos at Ol Jogi Wildlife Conservancy to determine if suboptimal reproductive performance was related to irregular cyclicity or anestrus, and if there were differences in endocrine activity between optimal and sub-optimal reproducing individuals.

## Methods

### Study site and study animals

Ol Jogi Wildlife Conservancy (Ol Jogi) in Laikipia County, Kenya is a fenced area of 235 km^2^ and was established as a rhinoceros sanctuary in 1980. The conservancy holds a population of 67 black rhinoceros—36 males, 28 females and 3 unsexed. Individual rhinos at Ol Jogi are identified by ear-notches, unique physical features and horn shapes and sizes enabling long-term monitoring of individuals. Of the 28 females on the property, 17 were of reproductive age (6–28 years) and all were included in the study (Table 1).

**Table 1:** Summary of reproductive history and endocrine data of reproductive female eastern black rhinoceros (*Diceros bicornis michaeli*) at Ol Jogi Wildlife Conservancy in Kenya

**Rhino I.D.**	**Age at start of study (years)**	**Date of first calf recorded**	**Date of last calving**	**Number of calves**	Inter-calving interval (years)*	**Reproductive success category**	**Sample collection period (months)**	**Number of estrous cycles**	**Estrous cycle length (days)**	Mean estrous cycle length (days)*	**Gestation periods (months)**	**Anestrous length (days)**
R-KY	28	21/01/2010	13/09/2018	4	2.88 ± 0.73	Optimal	12	2	39, 49	44 ± 5	7.5	77, 49
R-KL	27	3/10/2000	27/08/2016	7	2.65 ± 0.17	Optimal	10	0	0	0	10	0
R-TW	24	16/03/2004	17/12/2017	7	3.66 ± 0.47	Suboptimal	10	0	0	0	0	0
R-HL	20	03/11/2011	13/09/2017	3	2.88 ± 0.39	Optimal	7	2	21, 51	36 ± 15	0	65
R-MY	17	27/04/2013	21/09/2017	4	2.20 ± 0.12	Optimal	10	0	0	0		
R-MG	16	18/04/2011	06/12/2018	4	3.04 ± 0.51	Optimal	5	1	28	28	0	97
R-CR	15	26/06/2012	08/02/2016	2	3.62	Suboptimal	12	3	26, 31, 48	35 ± 7	7.5	44
R-JN	14	23/05/2014	23/05/2014	3	3.35 ± 0.32	Suboptimal	5	0	0	0	5	0
R-KS	14	18/11/2012	15/10/2016	4	1.95 ± 0.51	Optimal	10	0	0	0	10	0
R-CC	14	9/02/2014	23/02/2018	2	4.04	Suboptimal	5	2	N/E	N/E	0	45
R-TR	14	12/09/2012	02/10/2015	2	3.05	Optimal	12	2	47, 19	33 ± 14	3.5	41
R-SL	14	29/05/2014	05/07/2017	3	3.10 ± 0.21	Suboptimal	10	0	0	0	10	0
R-MN	14	14/03/2016	14/03/2016	1		NA	12	0	0	0	12	0
R-CH	9	29/07/2015	07/04/2017	3	1.69 ± 0.50	Optimal	10	0	0	0	10	0
R-JB	7	30/01/2018	01/03/2019	2	2.40	Optimal	10					
R-NM	7	Nulliparous				NA	4	2	45, 32	38.5 ± 6.5	0	0
R-EW	6	Nulliparous				NA	4	0	N/A		4	0
Overall*	15.33 ± 1.83			3.4 ± 0.45	2.89 ± 0.19		8.71 ± 0.86			35.7 ± 3.2		67 ± 13

*Mean ± SEM

N/E, not estimated

Reproduction was categorized as optimal or sub-optimal based on inter-calving intervals obtained from Ol Jogi’s historical reproductive database of field observations. Individuals with an average inter-calf interval of < 3 years were considered optimal, while those with an interval of > 3 years were considered sub-optimal (du Toit, 2006) (Table 1).

### Sample collection

Rhinos at Ol Jogi are monitored daily by rhino monitoring rangers to ascertain their safety and well-being. Fecal samples (50 g) were collected after observed defecations by rangers during patrols within 2 hours of defecation, at least twice a week for 5 to 12 months (average, 8.7 ± 0.7 months) between March 2019 and March 2020. March to June 2019 corresponded with a drought period, while August November 2019 were wet months. Samples were collected into ziploc bags, placed inside an insulated cool bag with a frozen icepack immediately after collection, and stored frozen at −20°C at Ol Jogi until transport to the laboratory at Mpala Research Centre.

### Laboratory analysis

Hormones were extracted from feces using an established wet weight vortexing method (Edwards *et al.,* 2014; Kamau *et al*. 2021). In brief, samples were thawed, thoroughly mixed, and 0.5 g (± 0.02) extracted with 90% methanol by vortexing for 30 minutes and centrifuging at 1800 g for 20 minutes. Supernatants were dried under air in a warm water bath, reconstituted with 1 ml of assay buffer (Cat. No. X065, Arbor Assays, Ann Arbor, MI USA), sonicated until completely re-suspended and then frozen at −20°C until analysis.

Fecal progestagen metabolites (fPM) were analysed by an enzyme immunoassay (EIA) (DetectX © Progesterone EIA K025, Arbor Assays, Ann Arbor, MI) that relies on a monoclonal progesterone antibody (CL425) validated for black rhinoceros (Edwards *et al.,* 2015). Fecal extracts were diluted in EIA buffer (1:100 for estrous cycles and 1:1000 during gestation) and analysed in duplicate. Serial dilutions of fecal extracts were parallel to the standard curve (R^2^ = 0.9458, y = 0.2087x + 1.3329; P < 0.05). Intra- and inter-assay coefficients of variation were < 10%.

## Data Analysis

Estrous cycles were characterized based on fPM using an iterative approach (Brown *et al.,* 2001; Edwards *et al.,* 2015). Baseline fPM concentrations were calculated by excluding values that exceeded the mean plus 1.5 standard deviations (SD) and continuing the elimination process until no values exceeded that cutoff. Onset of the luteal phase was defined as the first point above baseline that remained elevated for at least two consecutive points. The end of the luteal phase was defined as the first of two consecutive baseline values. Estrous cycle length was calculated as the beginning of one luteal phase to the beginning of the next. Irregular estrous cycles were defined as periods between the beginning and the end of a luteal phase that were longer than 40 or shorter than 20 days. Anestrus was defined as an interluteal period that exceeded twice the length of a normal follicular phase (i.e. > 10 days) (Brown *et al.,* 2001; Edwards *et al.,* 2015).

Seasonality was assessed by aligning periods of ovarian fPM activity, anestrus and irregular ovarian cycles with the month of the year and plotting bar graphs to determine if an observable pattern was present (Garnier *et al.,* 2002). We also used historical calving data from Ol Jogi to determine the distribution of births throughout the year. The prevalence of irregular estrous cycles was calculated by dividing the total number of irregular estrous cycles by the total number of estrous cycles observed from both optimal and suboptimal reproducing individuals. The prevalence of irregular estrous cyclicity was then compared between both optimal and sub-optimal reproducing females using a Wilcoxon rank sum test.

## Results

Baseline and average non-pregnant (NPLP) and pregnant (PLP) luteal phase fPM concentrations for each individual are summarized in Table 2. There was a wide range among rhinos in baseline concentrations (0.05–0.26 μg/g) fPM concentrations, with a 50-fold difference between low and high values. Peak concentrations during PLP and NPLPs also varied across individuals; e.g. < 2 μg/g (Fig. 3) to > 10 μg/g (Fig. 4). Overall PLP concentrations were just over 30% higher than NPLP (1.01–2.32 μg/g) concentrations and in general were more variable (0.28–1.84 μg/g) (Table 2). However, there was considerable overlap in PLP and NPLP concentrations within individuals, with the exception of R-TR where PLP concentrations were notably higher than NPLP values.

**Table 2:** Summary of baseline and mean (SEM) non-pregnant (NPLP) and pregnant (PLP) luteal phase concentrations of fecal progestagen metabolites (fPM) for individual reproductive female eastern black rhinoceros (*Diceros bicornis michaeli*) at Ol Jogi Wildlife Conservancy in Kenya

**Rhino I.D.**	**Baseline fPM (μg/g)** ^*^	**NPLP fPM (μg/g)** ^*^	**PLP fPM (μg/g)** ^*^
R-KY	0.05	0.81 ± 0.20	1.01 ± 0.22
R-KL	0.12	N/A	1.07 ± 0.14
R-TW	0.08	0.38 ± 0.07	N/A
R-HL	0.08	0.32 ± 0.06	N/A
R-MG	0.14	3.19 ± 1.09	N/A
R-CR	0.06	0.28 ± 0.04	1.47 ± 0.17
R-JN	0.13	N/A	1.93 ± 0.32
R-CC	0.26	1.00 ± 0.03	N/A
R-KS	0.11	N/A	1.69 ± 0.18
R-TR	0.15	1.84 ± 0.34	1.43 ± 0.20
R-SL	0.13	N/A	2.00 ± 0.13
R-MN	0.05	N/A	1.30 ± 0.15
R-CH	0.19	N/A	1.78 ± 0.18
R-NM	0.24	0.74 ± 0.18	N/A
R-EW	0.26	N/A	2.32 ± 0.41
**Overall**	**0.14 ± 0.01**	**1.07 ± 0.07**	**1.60 ± 0.03**

*Mean ± SEM

N/A, not applicable

**Figure 1:**
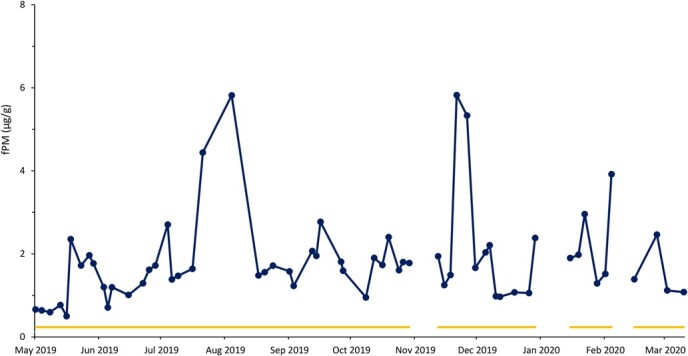
Fecal progestagen metabolite (fPM) profile for a pregnant female eastern black rhinoceros (*Diceros bicornis michaeli*) (R-SL) between May 2019 and March 2020 at Ol Jogi Wildlife Conservancy, with the mean + 1.5 SD cutoff value indicated by a yellow line

**Figure 2:**
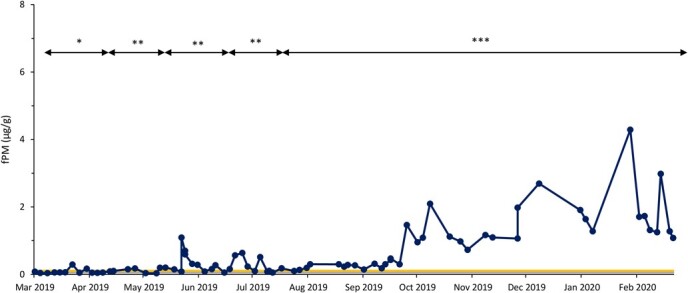
Fecal progestagen metabolite (fPM) profile for a female eastern black rhinoceros (*Diceros bicornis michaeli*) (R-CR) that showed estrous cyclicity followed by conception at Ol Jogi Wildlife Conservancy between March 2019 and March 2020. The mean + 1.5 SD cutoff value is indicated by a yellow line. Asterisks indicate pregnancy (***), estrous cycle (**) and anestrous (*) periods

**Figure 3:**
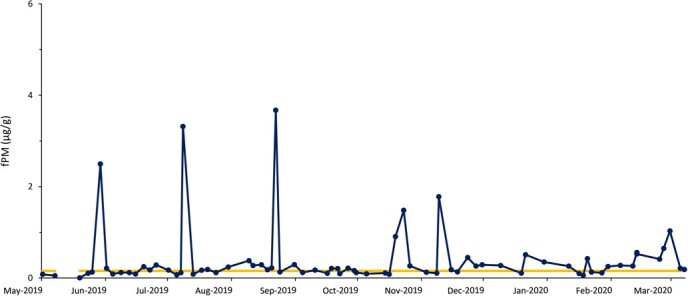
Fecal progestagen metabolite (fPM) profile of a female eastern black rhinoceros (*Diceros bicornis michaeli*) (R-TW) that exhibited fluctuations in fPM concentrations above the 1.5 S.D. cutoff (yellow line) between May 2019 and March 2020 that did not equate with either breeding or calving at Ol Jogi Wildlife Conservancy

**Figure 4:**
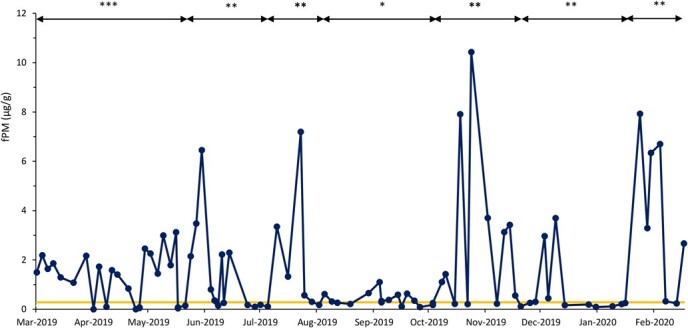
Fecal progestagen metabolite (fPM) profile for a female eastern black rhinoceros (*Diceros bicornis michaeli*) (R-HL) that showed elevated fPM concentrations above the mean + 1.5 SD (yellow line) at the beginning of sample collection period, followed by fPM concentration declines below baseline levels at Ol Jogi Wildlife Conservancy. Asterisks indicate pregnancy (***), estrous cycle (**) and anestrous (*) periods

Based on fPM analysis, all females exhibited ovarian activity, which included pregnancy (n = 10), cyclicity (n = 7) and anestrous (n = 6) periods (Table 1). Seven rhinos were pregnant throughout the sample collection period (Fig. 1), while two showed estrous cyclicity followed by conception (Fig. 2). Four rhinos exhibited estrous cyclicity throughout the sample collection period, while one rhino (Fig. 3) exhibited fluctuations in fPM concentrations above the 1.5 S.D. cutoff for the entire 10-month sampling period. One rhino exhibited elevated fPM concentrations for 76 days at the beginning of sample collection period, after which the concentrations declined to below baseline (Fig. 4). She then resumed cycling immediately thereafter up to the end of the sample collection period. This rhino's NPLP fPM concentration was higher than PLP fPM concentrations (Table 2).

A total of 12 complete cycles were observed from seven rhinos during the sample collection period, which ranged from 19–51 days with an average length of 36 ± 3 days. Half of the cycles were within the normal range, between.

20–40 days. Irregular cyclicity was also observed, including short (<20 days; 8.3%) and long (>40 days; 41.7%) cycles (Table 1).

**Figure 5:**
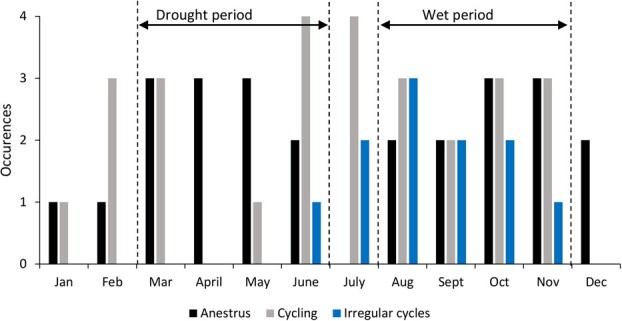
Bar graphs showing trends in the occurrence of normal ovarian cycles, irregular cycles and anestrous periods in eastern black rhinoceros (*Diceros bicornis michaeli*) females between March 2019 and March 2020 at Ol Jogi Wildlife Conservancy

**Figure 6:**
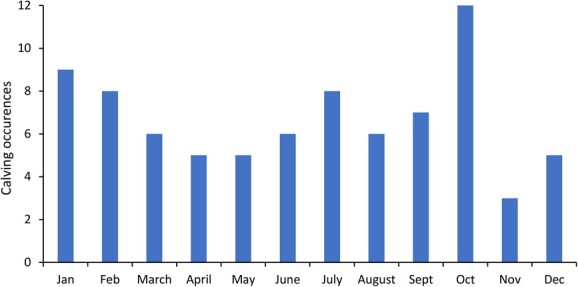
Bar graph showing calving trends from historical calving data of the eastern black rhinos (*Diceros bicornis michaeli*) between 1989 and 2020 at Ol Jogi Wildlife Conservancy.

Female rhinos exhibited ovarian cycles throughout the year, except for April 2019 and December 2019. The highest rates of ovarian cyclicity were observed between June and July, which corresponded to the end of a drought period (Fig. 5). Irregular cycles were also observed in June, but mostly occurred throughout the wet period (July–November). The rhinos in this study exhibited anestrus periods throughout the year except in July, which was between the drought and wet periods. Single anestrous periods were observed in five rhinos (e.g. Figs 2 and 3; Table 1), ranging from 41 to 128 days with an average of 67 ± 13 days. In general, a bimodal pattern of anestrus was evident, with more occurrences observed between March and May and October and November. Acyclic phases in three rhinos occurred between March and June 2019 (e.g. Fig. 4), corresponding to a period of drought in Laikipia, while two additional acyclic periods were observed during the wet season between August and November (e.g. Fig. 5). A clear pattern of births in relation to cyclicity status was not evident. Based on Ol Jogi records, births were observed in every month of the year, with lower occurrences in March to May (Fig. 6), corresponding to higher rates of anestrus (Fig. 5). However, births were highest in October, which also was associated with anestrus, followed by the lowest number the following month (November) that was similar in the occurrence of anestrus.

Of the six rhinos showing irregular cyclicity, four were categorized as having optimal reproduction, one was suboptimal and one was of reproductive age (7.24 years) but yet to calve (R-NM). Optimal reproducing individuals recorded a higher prevalence of irregular estrus cycles (66.7%) compared to the sub-optimally reproducing individuals (33.3%). Five rhinos exhibited anestrus during the sample collection period; two were categorized as suboptimal while the remaining three were considered optimal. Overall, the optimal reproducing individuals (69.5%) showed a higher prevalence of anestrous activity compared to suboptimal reproducing females (30.5%). However, due to limited animal numbers, there was no difference in the occurrence of irregular estrous cycles (Wilcoxon rank sum test W = 4, *p*= 0.6667) or anestrus periods (Wilcoxon rank sum test W = 2, *p* = 0.8) between the optimally and sub-optimally reproducing female eastern black rhino at Ol Jogi. Two females exhibiting anestrus also had been pregnant for part of the study period.

## Discussion

This study analysed fPM in 17 free-ranging female eastern black rhinos at Ol Jogi Wildlife Conservancy in Kenya. All exhibited variable ovarian activity including pregnancy (n = 10), normal ovarian cyclic activity (n = 7) and irregular cycles (n = 5). There was an observable pattern in the occurrence of anestrous periods and irregular cycles, both long (>40 days) and short (<20 days), suggesting that ovarian activity in this population may be influenced by prevailing weather conditions. Irregular cyclicity was observed in both optimal and sub-optimal reproducing females, although data was limited for further statistical analysis. We did note, however, that optimal reproducing females exhibited a higher prevalence of irregular estrous cycles and anestrus periods compared to sub-optimal reproducing females, suggesting these reproductive anomalies are not having significant impacts on reproductive success in this population.

Average estrous cycle lengths and fPM concentrations of rhinos at Ol Jogi were comparable to those of European and North American captive populations (Schwarzenberger *et al.,* 1993; Brown *et al.,* 2001 ; Edwards *et al.,* 2015), as well as those *in situ* (Garnier *et al.,* 2002; Freeman *et al.,* 2014). In this study, rhinos cycled throughout the year except for April and December, which were peaks of the dry and wet periods, respectively, in Laikipia in 2019. In Zimbabwe, optimum fertility coincided with the late spring and early summer, corresponding to the early rainy season (Garnier *et al.,* 2002). Irregular estrous cyclicity in Ol Jogi rhinos was observed towards the end of the dry season and throughout the wet period, with none observed between December and May. Anestrus periods were observed all year round, but also with a higher occurrence during both drought and wet periods. In zoo black rhinos, irregular estrous cycles and anestrus are not believed to be normal seasonal occurrences (Brown *et al.,* 2001; Edwards *et al.,* 2015), but may be influenced by adverse weather conditions and acute changes in prevailing weather conditions at Ol Jogi.

A 24-year-old multiparous rhino with a 1.2-year-old-calf at the beginning of the study was observed to have consistently elevated fPM concentrations throughout the 10-month sample collection period. Elevated progestagens have been reported in southern white rhinos (*Ceratotherium simum simum*) lasting up to 8 to 10 weeks due to uterine pathology associated with embryonic mortality or fetal resorption (Radcliffe *et al.,* 1997; Patton *et al.,* 1999). Elevated progestagen concentrations also have been associated with uterine pathology and persistent corpora lutea (Lueders *et al.,* 2018) and before death (Glaeser *et al.,* 2012) in Asian elephants, and so may serve as an indicator of distress associated with declining health. The cause of the 10-month duration of elevated fPM concentrations in this rhino is unknown as she appeared to be in good health. Future studies could strive to use noninvasive hormone monitoring in conjunction with opportunistic ovarian and uterine ultrasound examinations to document pathologies as possible causes of reduced fertility in free-ranging rhinos, particularly in isolated cases where the cause of elevated fPM concentrations is unknown.

A 14-year-old multiparous rhino with a 3-year-old calf at the beginning of the sampling period had elevated fPM concentrations for the first 76 days of the sampling period, after which the concentrations declined to below baseline. At this point, she was observed by the rhino monitoring rangers to exhibit calving behaviour by hiding in a thick patch of bush for several days but emerged without a calf. She then resumed cycling immediately thereafter and through the end of the sample collection period. Rhino fPM concentrations increase from the third month of gestation onward and exceed NPLP concentrations by approximately 10-fold from the seventh month of gestation onward (Edwards *et al.,* 2015). This rhino exhibited lower fPM, and so, while the date of conception was not known, the absence of a calf/carcass and high fPM concentrations may be indicative of fetal loss. Isolated cases of early fetal loss have been reported in wild (Garnier *et al.,* 2002) and captive (Berkeley *et al.,* 1997) black rhino, as well as in the three other rhino species (Radcliffe *et al.,* 1997; Roth *et al.,* 2001: Stoops *et al.,* 2014), although prevalence and causes are unknown. Use of non-invasive hormone monitoring will be key in identifying whether early fetal losses are a common occurrence and their cause which will guide interventions. Thus, use of non-invasive hormone monitoring will be key to identifying whether early fetal loss is a common occurrence in this and other black rhino populations.

A majority of estrus cycles in the European eastern black rhino population were categorized as normal (20–40 days long), with 12.4% being short and 14.7% being long (Edwards *et al.,* 2015). In North America, 61% were normal, with 21% being longer and 18% being shorter (Brown *et al.,* 2001). In a study of free-ranging black rhino in Zimbabwe, Garnier *et al.* (2002) found three-quarters of cycles were within the normal range, while the rest were longer and due to either extended follicular or luteal phases. At Ol Jogi, 50% of estrous cycles were normal, 8.3% were short (<20 days) and 41.7% were long. In Northern America and European zoos, conception is not associated with the occurrence of irregular estrous cycles in rhinos (Brown *et al.,* 2001; Edwards *et al.,* 2015). In this study, conception occurred after the occurrence of long estrous cycles in two rhinos, suggesting that irregular ovarian activity in free-ranging eastern black rhinos does not affect fertility. Although animal numbers were limited, this idea is supported by the observation of regular and irregular estrous cyclicity in both optimal and sub-optimal reproducing individuals.

The yearly percentage of females calving is an important measure of population performance and assumes all females ≥7 years old should be reproducing (du Toit, 2006). Because of the 15- to 16-month gestation period, the yearly percentage of females calving fluctuates on an yearly basis and calving rates are generally averaged over ≥3-year periods (Okita-Ouma *et al.,* 2021). Whereas we aimed to determine whether suboptimal reproductive performance may be due to erratic cyclicity and/or pregnancy losses, we undertook this study just before a calving boom was observed at Ol Jogi (four-fold increase compared to the preceding year) and also at Lewa Wildlife Conservancy and Meru National Park in Kenya (KWS, 2020; Koech, 2020). Thus, most individuals were pregnant (n = 10) with ovarian cyclicity data obtained from just seven individuals.

In summary, assessment of fecal progestagen metabolite profiles in free-ranging female eastern black rhino in Kenya is just a first step to better understanding factors influencing their reproductive success. There were some limitations to this study. First, due to the cryptic nature of the Eastern black rhino, there were sample collection gaps and so the length of some estrous cycles could not be calculated. Second, our intention was to collect samples for a 12-month period from the 17 reproductive aged rhinos at Ol Jogi; however, sample collection was discontinued in March 2020 due to the Covid-19 pandemic. Thus, results on ovarian cyclicity are from few individuals assessed for varied and in some cases short periods of time, so we recommend that future studies of this subspecies be conducted for longer periods (at least 2 years) to capture any seasonal effects on ovarian function. Still, overall results suggest that irregular ovarian activity does not appear to be having an impact on reproductive success in free-ranging eastern black rhino. A high priority must be to use noninvasive hormone (reproductive and adrenal) monitoring of both males and females in conjunction with collecting data on ecological and other physiological variables, such as sex ratios, competing browser populations, distance to water sources, home range size and drought and rainfall patterns, to determine the impact of both intrinsic and extrinsic factors on individual and population measures of reproductive success. Hormone monitoring in free ranging eastern black rhino also can be used to inform decisions on individuals fit for translocation and to monitor how rhinos acclimatize to new sites. In that regard, it can serve as an important conservation tool to better manage Kenya’s vulnerable rhino subpopulations. Scaled up, the newly established endocrine laboratory at Mpala can also inform on factors affecting reproductive performance of other endangered species in the region.

## Funding

This study was funded by a National Geographic Exploration Grant (NGS-59622R-19). The Smithsonian’s veterinary operations in Kenya is also supported by a grant from the Smithsonian Women’s Committee. Core support for the Smithsonian Conservation Biology Institute’s Global Health Program’s training programme is provided by Dennis and Connie Keller.

## Conflicts of interest

The authors have no conflicts to declare.

## Data availability

The data underlying this article will be shared on reasonable request to the corresponding author.

## Author contributions

The authors confirm contribution to the paper as follows: study conception and design: Maureen W. Kamau, Janine L. Brown, Jamie Gaymer, James Hassell, Dino J. Martins, Suzan Murray; assisted acquire funding for the study: Maureen W. Kamau, Dino J. Martins, Suzan Murray; data collection: Maureen W. Kamau, Jamie Gaymer, James Hassell; Laboratory analysis of samples collected: Nicole Boisseau, Maureen W. Kamau; analysis and interpretation of results: Maureen W. Kamau, Janine L. Brown, Suzan Murray; draft manuscript preparation: Maureen W. Kamau, Janine L. Brown, Suzan Murray. All authors reviewed the results and approved the final version of the manuscript.
